# A Novel Human Neutralizing mAb Recognizes Delta, Gamma and Omicron Variants of SARS-CoV-2 and Can Be Used in Combination with Sotrovimab

**DOI:** 10.3390/ijms23105556

**Published:** 2022-05-16

**Authors:** Margherita Passariello, Veronica Ferrucci, Emanuele Sasso, Lorenzo Manna, Rosa Rapuano Lembo, Stefano Pascarella, Giovanna Fusco, Nicola Zambrano, Massimo Zollo, Claudia De Lorenzo

**Affiliations:** 1 Ceinge—Biotecnologie Avanzate s.c.a.r.l., Via Gaetano Salvatore 486, 80145 Naples, Italy; margherita.passariello@unina.it (M.P.); veronica.ferrucci@libero.it (V.F.); emanuele.sasso@unina.it (E.S.); lorenzo.manna@unina.it (L.M.); rosa.rapuano@unimi.it (R.R.L.); zambrano@unina.it (N.Z.); mzollo99@gmail.com (M.Z.); 2 Department of Molecular Medicine and Medical Biotechnology, University of Naples “Federico II”, Via Pansini 5, 80131 Naples, Italy; 3 European School of Molecular Medicine, University of Milan, 20122 Milan, Italy; 4 Department of Biochemical Sciences “A. Rossi Fanelli”, Sapienza University of Rome, Viale Regina Elena 332, 00185 Rome, Italy; stefano.pascarella@uniroma1.it; 5 Istituto Zooprofilattico Sperimentale del Mezzogiorno, Via Salute 2, 80055 Portici Naples, Italy; giovanna.fusco@izsmportici.it

**Keywords:** SARS-CoV-2, Omicron variant, neutralizing mAbs, Spike-RBD/ACE-2, combinatorial treatments

## Abstract

The dramatic experience with SARS-CoV-2 has alerted the scientific community to be ready to face new epidemics/pandemics caused by new variants. Among the therapies against the pandemic SARS-CoV-2 virus, monoclonal Antibodies (mAbs) targeting the Spike glycoprotein have represented good drugs to interfere in the Spike/ Angiotensin Converting Enzyme-2 (ACE-2) interaction, preventing virus cell entry and subsequent infection, especially in patients with a defective immune system. We obtained, by an innovative phage display selection strategy, specific binders recognizing different epitopes of Spike. The novel human antibodies specifically bind to Spike-Receptor Binding Domain (RBD) in a nanomolar range and interfere in the interaction of Spike with the ACE-2 receptor. We report here that one of these mAbs, named D3, shows neutralizing activity for virus infection in cell cultures by different SARS-CoV-2 variants and retains the ability to recognize the Omicron-derived recombinant RBD differently from the antibodies Casirivimab or Imdevimab. Since anti-Spike mAbs, used individually, might be unable to block the virus cell entry especially in the case of resistant variants, we investigated the possibility to combine D3 with the antibody in clinical use Sotrovimab, and we found that they recognize distinct epitopes and show additive inhibitory effects on the interaction of Omicron-RBD with ACE-2 receptor. Thus, we propose to exploit these mAbs in combinatorial treatments to enhance their potential for both diagnostic and therapeutic applications in the current and future pandemic waves of coronavirus.

## 1. Introduction

On January 2020, a novel pneumonia virus, expanding abruptly worldwide, was identified and named 2019 novel coronavirus (2019-nCoV) [1,2] or SARS-CoV-2. The latter is a highly transmissible virus, which mainly affects the respiratory system, by infecting the cells through the Angiotensin Converting Enzyme-2 (ACE-2) in human hosts, which binds to the Spike-Receptor Binding Domain (RBD) protein of the coronavirus. This binding is crucial for facilitating the cell viral entry into the host cells [3,4,5]. The emergence of SARS-CoV-2 was characterized by a period of relative evolutionary stasis followed by a continuous emergence of novel mutations in the SARS-CoV-2 genome that have affected important functional properties of the virus, such as its infectivity, transmissibility, antigenicity or disease severity. 

The mapping of the SARS-CoV-2 genome and the investigation on sequence variations, available in a sequence database [6,7], ensure mutations tracking by quickly identifying the new SARS-CoV-2 variants that accounted for 90% of new infections in some regions. To date, the World Health Organization (WHO) has identified five SARS-CoV-2 variants of concern (VOC): Alpha (B.1.1.7), Beta (B1.351), Gamma (P.1), Delta (D.1) and Omicron (B.1.1.529), and two SARS-CoV-2 variants of interest (VOI), Lambda (C.37) and Mu (B.1.621) [8]. Adescription of positive advantages of selective mutations occurring in emerging clades was recently described in a pandemic COVID-19 virus tracing analysis in Italy [9].

The Omicron variant, first identified in November 2021 in South Africa, and three Omicron subvariants classified as BA.1, BA.2 and BA.3 are diffusing worldwide, becoming predominant over the others. Although the main Omicron subvariant BA.1 seems to have reduced severity of clinical outcomes and risk of hospitalization, when compared with the Delta variant, it has a much higher incidence rate due to its marked contagiousness [10]. BA.1 is characterized by the emergence of over 30 mutations in the Spike protein promoting its cell entry as well as its potential to develop immune escape mechanisms, thus evading the antibodies produced after vaccination or previous COVID-19 infections [11,12].

About 15 of these mutations occur in the RBD region of Spike, affecting the binding free energy (BFE) change of the Spike-RBD/ACE-2 complex, which influences the virus infectivity [13]. Some mutations found in the other emerged variants were involved in the binding affinity of viral ligands for their cellular receptors, rising cell infection and disease severity; other mutations, such as those present in Beta and Gamma variants, decreased the affinity of RBD for ACE-2 and facilitated the immune escape [14]. These findings suggest a cooperative effect between mutations that could enhance immune escape, leading to a decreased efficacy of therapeutic options.

The design and discovery of therapeutic monoclonal antibodies (mAbs) are part of an important achievement in fighting cancer and viral diseases [15], including COVID-19; indeed, mAbs targeting the RBD domain, or other epitopes of the Spike protein, are commonly used to neutralize the virus infection [16,17] but their effectiveness could be significantly affected by newly emerging variants. 

Five anti-SARS-CoV-2 mAbs received the Emergency Use Authorizations (EUAs) from the Food and Drug Administration (FDA) for the treatment of COVID-19: Bamlanivimab plus Etesevimab, Casirivimab plus Imdevimab (REGEN-COV), and Sotrovimab (GlaxoSmithKline and Vir Biotechnology) [18]. Specifically, therapies based on these mAbs, used often in combinations, have been approved for the treatment of mild to moderate COVID-19 in non-hospitalized patients who are at high risk for progressing to severe disease. Nevertheless, since the new Omicron variant has emerged, the efficacy of the treatments with Bamlanivimab plus Etesevimab or Casirivimab plus Imdevimab decreased, which was likely because Omicron subvariants have multiple mutations in the Receptor-Binding Motif (RBM) recognized by all these mAbs [19]. 

Only the GlaxoSmithKline’s mAb Sotrovimab retains the activity also against the Omicron variant, and it is the only antibody–drug authorized in the U.S. for the treatment of COVID-19 patients infected by Omicron [12,18,20,21]. 

Recently, we isolated a novel neutralizing anti-Spike human monoclonal antibody, named D3, by an innovative phage display strategy based on the competitive elution of phage antibodies from immobilized RBD with ACE-2. D3 was found to be able to specifically bind to the RBD domain in a nanomolar range and to interfere in the interaction of Spike protein with ACE-2 receptor, which was either used as a purified protein or when expressed on cells in its native conformation [22]. The D3 antibody efficiently inhibited the entry of the SARS-CoV-2 virus into target cells by totally neutralizing its infectivity, and it was also effective against its Alpha (B.1.1.7) variant, showing no side toxic or pro-inflammatory effects [22,23].

Thus, we decided to investigate the ability of the novel D3 mAb to inhibit the infection of other emerged variants such as Delta and Gamma as well as on its binding to the last predominant Omicron variant-derived Spike protein. To this aim, we firstly investigated the epitope of Spike-RBD protein recognized by D3, and then, we combined it with Sotrovimab to test their synergistic or additive effects on the Omicron-RBD/ACE-2 interaction in order to potentiate their efficacy through the use of novel combinatorial treatments.

## 2. Results

### 2.1. Epitope Mapping of D3 on Spike of SARS-CoV-2 by Testing Its Binding to Different Peptides Derived from the RBD Domain

**Figure 1:**
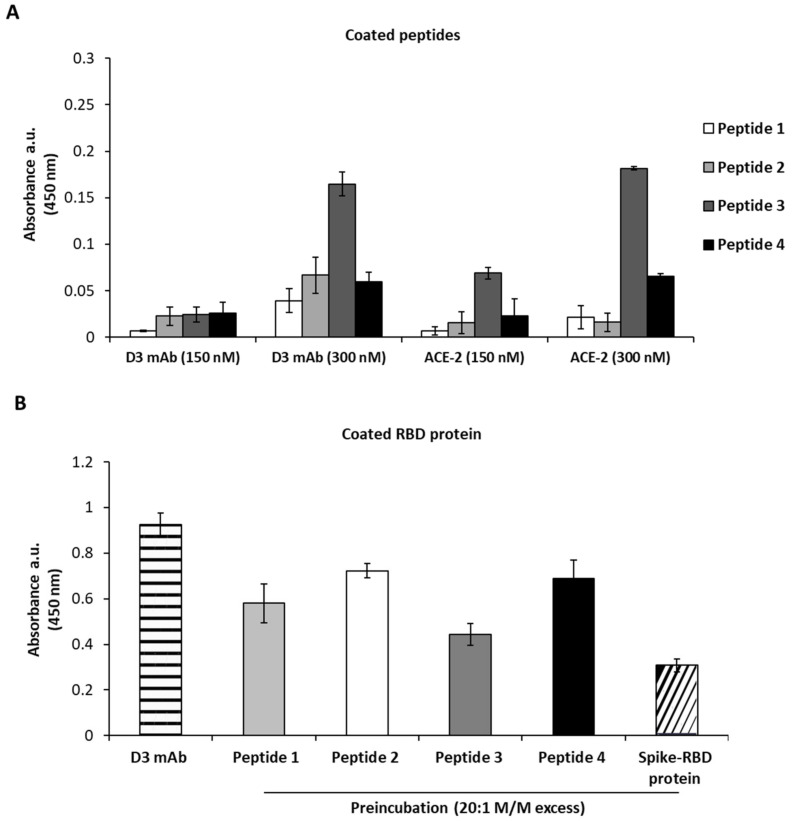
ELISA assays to test the binding of D3 to different peptides derived from Spike-RBD domain. (**A**) The peptides were immobilized on a 96-well plate at the concentration of 5 µg/mL and incubated with D3 mAb or ACE-2 receptor at the concentration of 150 and 300 nM. (**B**) The binding of D3 was detected on an immobilized RBD protein in the absence (striped bar) or in the presence of peptide 1 (light gray bar), peptide 2 (white bar), peptide 3 (dark gray bar), peptide 4 (black bar) or Spike-RBD protein (slashed bar), used at 20:1 molar excess with respect to mAb. Binding values were reported as the mean of at least three determinations. Error bars depicted means ± SD.

**Figure 2:**
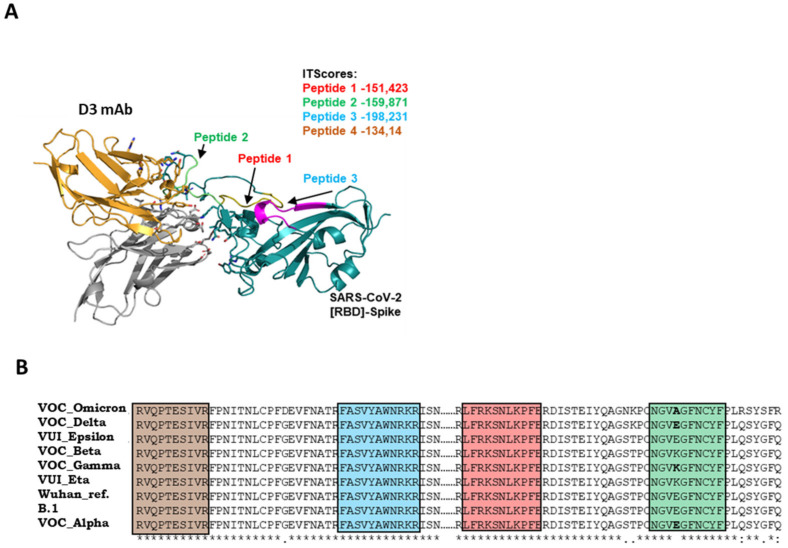
Molecular docking of D3 antibody with Spike protein of SARS-CoV-2. (**A**) The heavy and light chains of the D3 antibody are represented in yellow and gray, respectively. The SARS-CoV-2 Spike protein (RBD region) is presented in cyan. The first 3 peptides, as designed to map the interaction region between the D3 antibody and Spike protein, are also shown in red, green and blue, respectively. The fourth peptide is not shown because it is outside RBD region. The IT scores of the peptides are shown. (**B**) The aminoacidic sequences of the indicated Spike-RBD variants are reported by highlighting in colored squares those referred to the peptide 1 (red), peptide 2 (green), peptide 3 (blue) and peptide 4 (brown).

To investigate the epitope recognized by D3 on Spike, four different peptides derived from the sequence of the RBD domain were designed and synthetized in order to cover both RBM and non-RBM regions. ELISA assays were performed by testing the binding of D3 at increasing concentrations on these immobilized peptides (indicated as 1–4) in comparison with the ACE-2 receptor, which was used as a control in parallel assays. As shown in Figure 1A, we found that D3 specifically recognizes the peptide 3 (located in a region upstream to the RBM domain) in a similar fashion to ACE-2, which was used as a positive control. However, as expected, the binding affinity of both D3 and ACE-2 for the peptides is much lower than that observed for the whole RBD, and it is detectable only at high concentration (300 nM). To confirm these data, another method based on competitive ELISA assays was performed by measuring the binding of D3 mAb to immobilized Spike-RBD protein in the absence or in the presence of 20:1 molar excess of each peptide or the parental RBD, which was used as a positive control. As reported in Figure 1B, a significant reduction in the binding signal of D3 to RBD was observed in the presence of peptide 3, which was already found to be the preferential binding site in the previous experiment. These data are in line with the docking analyses of the complex of D3 with RBD, as shown in Figure 2, where the peptides used for the ELISA assays are evidenced both in the primary and in the tertiary structure of RBD.

### 2.2. Binding of D3 to Recombinant RBD of Omicron Variant

**Figure 3:**
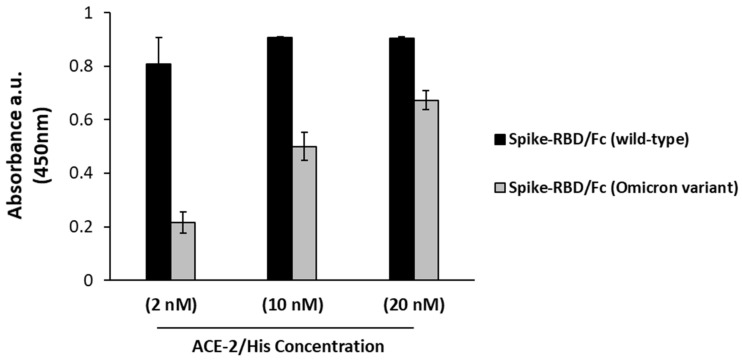
Binding of ACE-2 to Spike-RBD from wild-type SARS-CoV-2 or its Omicron variant. The binding of ACE-2 receptor to immobilized recombinant wild-type (black bars) or to Omicron BA.1 (gray bars) Spike-RBD was tested by using increasing concentrations of ACE-2/His followed by detection with anti-His-horseradish peroxidase (HRP)-conjugated antibody.

Recent data in the literature have reported a slight reduction in the binding of ACE-2 to the Omicron variant with respect to the wild-type Spike protein [19]. We firstly analyzed by ELISA assays the interaction of ACE-2 receptor with the Spike-RBD Omicron protein by testing the binding of recombinant ACE-2/His to both the recombinant wild-type or BA.1 subvariant Spike-RBD domains. The results reported in Figure 3 confirm that ACE-2 shows a lower affinity (5–10 fold) for the Omicron variant [9]. 

This result can be explained by the multiple mutations that have occurred in the receptor-binding motif of SARS-CoV-2 generating a number of different variants over the time, such as the last predominant Omicron variant, which is not recognized anymore by the available antibodies in clinical use [11,13]. An exception is represented by Sotrovimab mAb (GlaxoSmithKline and Vir Biotechnology) that was found to retain activity also against the Alpha, Beta, Gamma, Delta, and Lambda variants and to reduce the risk of disease progression [19,20]. 

**Figure 4:**
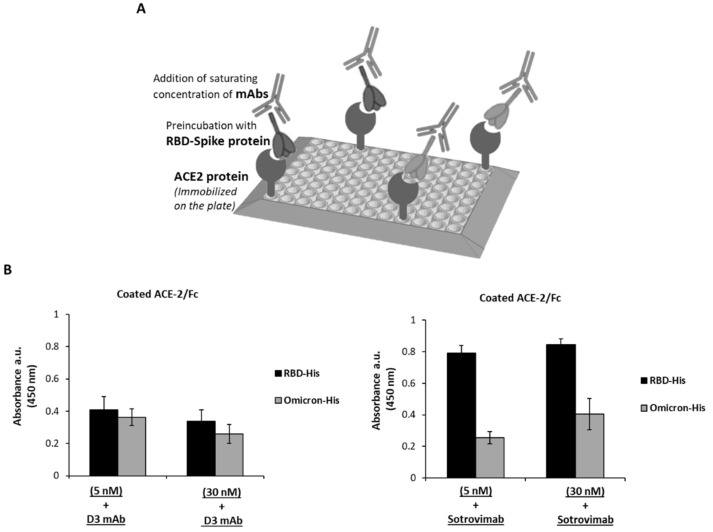
Comparison of the binding of D3 and Sotrovimab mAb to RBD-SARS-CoV-2 protein or its Omicron variant by ELISA assays. (**A**) Graphical representation of the ELISA assay to test the binding of D3 to the RBD proteins. (**B**) ACE-2/Fc recombinant protein was immobilized on the plate and pre-incubated with rRBD-SARS-CoV-2/His or rRBD-Omicron/His variant protein at concentrations of 5 nM or 30 nM. D3 or Sotrovimab mAb were added and detected by an anti-Fab HRP-conjugated antibody for their binding either to the parental RBD (black bars) or the Omicron variant (gray bars). The absorbance values were reported as the mean of at least three determinations. Error bars depicted means ± SD.

We tested the novel neutralizing anti-Spike mAb, D3, generated in our laboratory [22], for its binding to the BA.1 Omicron subvariant in comparison with Sotrovimab. To this aim, the ACE-2 receptor was immobilized on a 96-well Nunc plate and then incubated with the wild-type Spike-RBD protein or its BA.1 Omicron variant at the concentrations of 5 and 30 nM. The following binding of D3 or Sotrovimab mAb to the two RBD domains was detected by a secondary anti-Fab HRP-conjugated antibody, as shown in Figure 4B. The binding ability of D3 to the Omicron variant seems only partially decreased with respect to that observed on the parental protein, whereas Sotrovimab shows a more marked loss in its binding to Omicron subvariant with respect to that observed on the parental Spike protein. These findings confirm that D3 binds to a region of the RBD which is unmutated in the BA.1 Omicron subvariant.

### 2.3. Comparison of Binding to Omicron Variant of D3 and Other Therapeutic mAbs in Clinical Use

It is well known that anti-SARS-CoV-2-validated mAbs in clinical use in the first days post infection have been used in combination to increase their anti-viral potency [4]. For instance, two of the known neutralizing mAbs, such as Casirivimab and Imdevimab (Regeneron Pharmaceuticals), had been administered as combination therapy for the variants that are susceptible to these treatments [17]. However, the FDA revised this authorization following Omicron’s spread [18,19] exclusively confirming the authorization of Sotrovimab for the treatment of COVID-19 patients infected by the Omicron variant [17,18]. Thus, we decided to compare the binding ability of the novel D3 mAb to the Omicron variant with that of Sotrovimab by using Casirivimab or Imdevimab in parallel assays. A human unrelated IgG was also included in parallel assays as a negative control.

**Figure 5:**
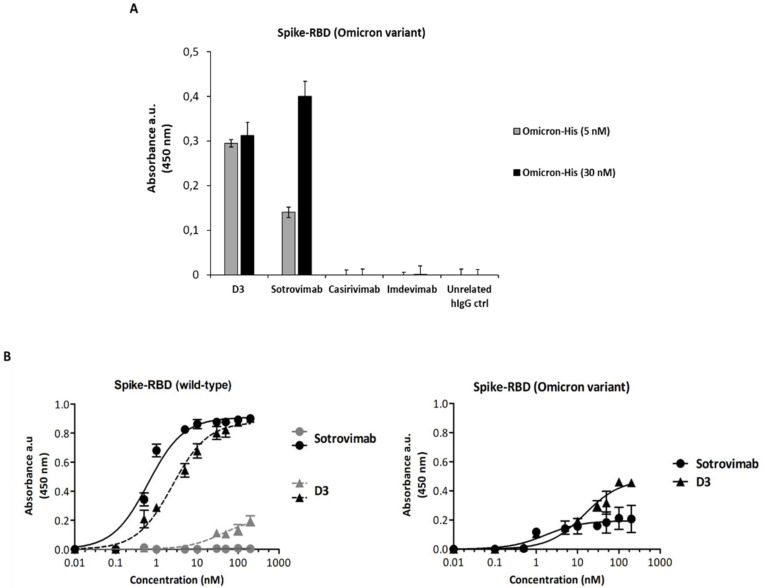
Binding comparison of D3 mAb to rRBD-SARS-CoV-2 Omicron variant with respect to that of Sotrovimab, Casirivimab or Imdevimab. (**A**) ACE-2/Fc recombinant protein was immobilized onto the plate and pre-incubated with rRBD-Omicron/His variant protein at concentrations of 5 nM (gray bars) or 30 nM (black bars). D3 or the other indicated mAbs were added and detected by an anti-Fab HRP-conjugated antibody for their binding to Omicron BA.1 subvariant. (**B**) D3 or Sotrovimab were incubated at increasing concentrations (0.5–200 nM) on immobilized recombinant Spike-RBD from wild-type SARS-CoV2 (left) or its Omicron variant (right). The gray curves show the binding of D3 (gray triangles) or Sotrovimab (gray circles) to the Fc domain, used as a negative control, the black curves show the binding of D3 (black triangles) or Sotrovimab (black circles) to each indicated Spike-RBD-Fc domain. Binding values were reported as the mean of at least three determinations. Error bars depicted means ± SD.

The experiment was performed as described above, and the results, shown in Figure 5A, indicate that D3 binds to the Omicron variant at nanomolar concentrations, whereas Casirivimab and Imdevimab are not able to bind to Omicron at these low concentrations, confirming previous observations about the lack of efficacy of these mAbs for Omicron infections [18,19]. 

To further investigate the binding affinities of D3 and Sotrovimab for the Omicron-derived RBD, we tested them in parallel assays at increasing concentrations either on purified recombinant wild-type Spike-RBD/Fc protein or on its Omicron BA.1 subvariant. The binding curves reported in Figure 5B show that both D3 and Sotrovimab seem to retain a significant binding to the mutant Omicron-RBD, even though a decrease of about 50–60% of the maximum absorbance value was observed for D3, and a more marked decrease (about 80%) was detected for Sotrovimab.

### 2.4. Competitive ELISA Assays to Verify Whether D3 and Sotrovimab mAbs Recognize Different Epitopes

**Figure 6:**
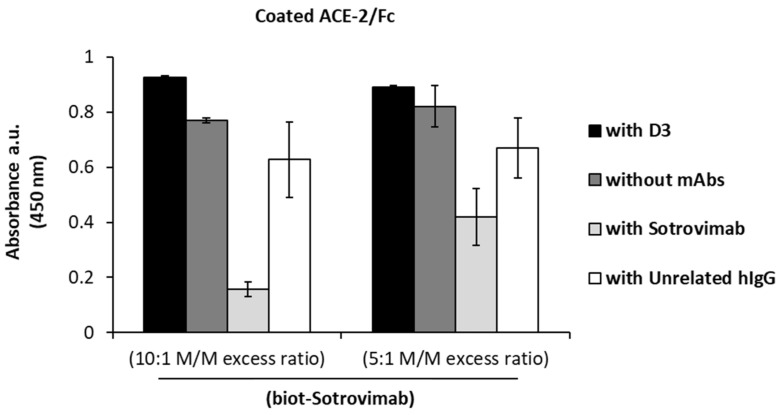
Competitive ELISA assays of D3 or Sotrovimab with Biotinylated Sotrovimab (biot-Sotrovimab). The ELISA assays were performed by pre-incubating the ACE-2 coated plate with RBD-SARS-CoV-2/His chimeric protein. The binding of the biot-Sotrovimab to RBD protein was measured in the absence of mAbs (dark gray bars) or in the presence of unlabeled-D3 (black bars), Sotrovimab (light gray bars) or unrelated (white bars) mAb (used at saturating concentration of 150 nM).

Since D3 and Sotrovimab mAbs are both able to recognize the Omicron variant, we further investigated the possibility to use them in combinatorial treatments. To this aim, we tested the two mAbs in a competitive ELISA assay to verify whether they compete for the binding to the wild-type Spike-RBD protein. The recombinant Spike-RBD was added to immobilized ACE-2 receptor, and then, the binding of biotinylated Sotrovimab (biot-Sotrovimab) was measured in the absence or in the presence of a molar excess (10:1 or 5:1) of unlabeled D3 or Sotrovimab mAb. The binding of biotinylated Sotrovimab was detected by HRP-streptavidin. As shown in Figure 6, the binding of biot-Sotrovimab to RBD is not significantly reduced in the presence of D3 mAb, or an unrelated hIgG, which was used as a negative control, whereas, as expected, it is efficiently competed by the unlabeled Sotrovimab, which was used as a positive control. These results indicate that the two mAbs recognize different epitopes in the RBD domain of Spike protein, as also suggested by previous reports on the epitope recognized by Sotrovimab [20], and accordingly, they could be used in combinatorial treatments in order to obtain more potent anti-viral activity.

**Figure 7:**
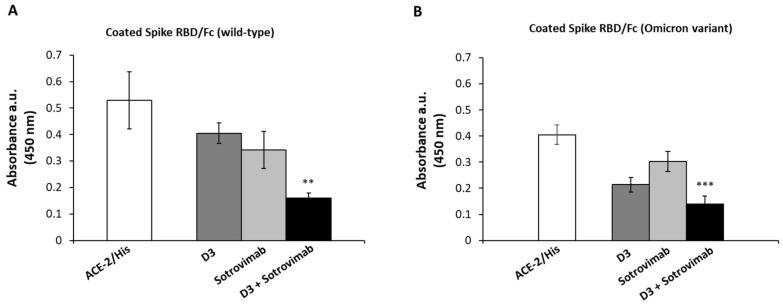
Competitive ELISA assays to test the interference of anti-Spike mAbs in the Spike/ACE-2 interaction. (A–B) The assays were performed by measuring the binding of ACE-2 receptor (5 nM) to recombinant wild-type (**A**) and to Omicron (**B**) Spike-RBD in the absence (white bars) or in the presence of D3 (dark gray bars) Sotrovimab (light gray bars) used alone, or their combination (black bars). The binding values were reported as the mean of at least three determinations. Error bars depicted means ± SD. P-value: *** *p* ≤ 0.001; ** *p* < 0.01.

As previously reported, both D3 and Sotrovimab were found to be able to interfere in the Spike/ACE-2 interaction [20,22]. Considering the results on the distinct epitopes recognized by the two mAbs, we tested whether the combination of D3 mAb with Sotrovimab can increase the interference in the interaction of Spike-RBD (both from wild-type Coronavirus or BA.1 Omicron subvariant) with the ACE-2 receptor with respect to single treatments. To this aim, ELISA assays were performed in parallel on the two recombinant immobilized RBD domains by measuring the binding of ACE-2 in the absence or in the presence of molar excess (10:1 M/M) of D3 or Sotrovimab mAb, respectively, or their combination. The results, shown in Figure 7A,B, indicate that the combination of the two mAbs reduces the binding of ACE-2 to the wild-type RBD and to the Omicron RBD by about 70% and 60%, respectively, showing an improvement with respect to the effects obtained by using D3 or Sotrovimab as single agents (20 or 30% inhibition in the case of parental RBD and 50% or 20% in the case of Omicron, respectively).

### 2.5. SARS-CoV-2 Variants Neutralization by the Novel Human mAb D3

To test the neutralizing activity of D3 against other variants, we investigated its efficacy on another two previously emerged variants of SARS-CoV-2 [8,9]. To this aim, the antibody (10 μg/mL) was preincubated with the viral particles of two different variants (VOC Delta, 0.01 MOI or VOC Gamma (i.e., P.1), 0.3 MOI) for 2 h at 37 °C. Then, the mixtures were added to primary human epithelial cells and incubated for 72 h at 37 °C.

**Figure 8:**
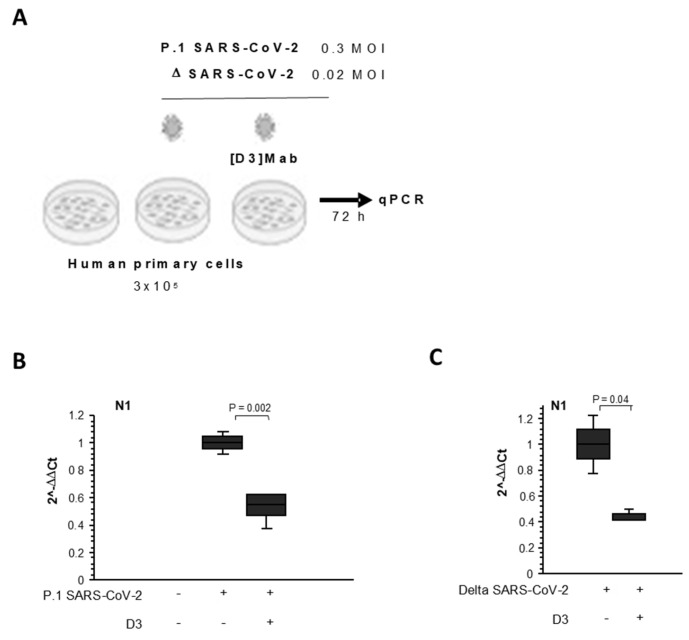
Effects of D3 on infectivity of Delta and Gamma variants in cell cultures. (**A**) Schematical representation of neutralization assays. Primary human epithelial cells from nasal brushing (3 × 10^5^) were plated in a 6-well plate and infected with SARS-CoV-2 viral particles (VOC delta, 0.02 MOIor VOC gamma (i.e., P.1), 0.3 MOI) previously incubated with D3 antibody for 2 h at 37 °C. Non-infected cells were used as the negative infection control. After 72 h, the cells were lysed, and their RNA was extracted. (**B**,**C**) Quantification of mRNA abundance relative to that in CTR cells (2−DDCt) of viral N gene from Real-Time PCR analysis with SYBR Green. Non-infected cells and SARS-CoV-2–infected cells treated with vehicle (CTR) were used as controls. Data are means ± SD. Statistical significance was defined as *p* <0.05 by unpaired two-tailed Student’s *t*-tests.

After the incubation, the cells were lysed, their RNA was extracted, and viral N gene was quantified by Real-Time PCR analysis with SYBR Green. As shown in Figure 8, D3 was able to efficiently inhibit the infection of human cells by both variants.

## 3. Discussion

The novel SARS-CoV-2 virus is spreading at an extremely fast pace worldwide, leading to the rapid generation of new variants, which show resistance to the available mAb-based therapies [1,2,8]. In particular, the efficacy of mAbs targeting the RBM domain of the Spike protein, approved for the treatment of COVID-19 [18], has been found decreased (with the exception of Sotrovimab) especially in the case of the Omicron variant [19,20,21].

In this study, we investigated on the ability of the novel human anti-Spike-RBD neutralizing D3 mAb, recently isolated in our laboratory and foundto be already effective on the Alpha variant of SARS-CoV-2 [22,23], to exert its anti-viral efficacy against some other variants of SARS-CoV-2, such as Delta, Gamma and Omicron. 

Firstly, we identified the epitope recognized by D3 by performing docking analyses and ELISA assays on four synthetic peptides derived from the RBD region, covering both RBM and non-RBM regions. Since the epitope seems to be upstream of the RBM, we tested whether D3 retains its binding to the RBD from Omicron subvariant. We found that the novel mAb efficiently recognizes the Omicron-derived recombinant RBD in in vitro assays, differently from the antibodies Casirivimab or Imdevimab, developed by Regeneron [19,24]. The latter two mAbs are likely susceptible to some specific Omicron mutations in the RBD, such as the E484A, S477N and T478K (in the RBM region) for Casirivimab and G446S (in the RBM region) for Imdevimab, whereas Sotrovimab could be only partially influenced by the N440K mutation (in the RBM region), even though it still preserves a significant affinity for the Omicron RBD [25].

We then investigated whether D3 and Sotrovimab mAbs competed for the binding to Spike-RBD, and we found that Sotrovimab binding was not reduced by the presence of saturating concentrations of D3, thus confirming that the two mAbs recognize different epitopes, as also suggested by the different sequences of peptide 3 recognized by D3 and those recognized by Sotrovimab and previously reported in the literature [18,20]. Subsequently, to test whether the novel D3 antibody could be used in combination with the neutralizing mAb Sotrovimab, the only antibody–drug currently authorized by the FDA for the treatment of COVID-19 patients infected by the last emerged RBD-Omicron variant, ELISA assays were performed by measuring the binding of D3 and Sotrovimab to the wild-type or its Omicron variant Spike-RBD. Surprisingly, D3 retained a higher binding capacity for Omicron-RBD, while Sotrovimab displayed a much more marked decrease in its affinity for the Omicron variant with respect to the parental protein. 

Our studies confirm that the mutations leading to the Omicron variant do not affect the epitope recognized by D3 (non-RBM) for the binding to the Spike-RBD protein; therefore, its neutralizing effectiveness should not be impaired. Thus, we evaluated the possibility to combine the two agents to increase their interference activity in Spike-RBD/ACE-2 interaction. We found that the two mAbs show additive effects in the binding interference between the two partners, as the combinatorial treatments of the two mAbs reduced by about 70% ACE-2/Spike-RBD binding, whereas only ≈30–50% of inhibition was observed when they were used as single mAbs. 

We finally investigated the neutralizing activity of D3 on the infectivity of other emerged variants of SARS-CoV-2 [8,9] by incubating the mAb with viral particles of Delta and Gamma variants before their infection of primary human epithelial cells, and we found that D3 fully retains its neutralizing efficacy against these variants, thus efficiently inhibiting their infectivity in cell cultures.

Recently, an additional variant has been identified, generated from the recombination of the BA.1 and BA.2 subvariants, which has been found in patients co-infected with the two variants. However, the recombination site does not involve the epitope recognized by D3, suggesting that the mAb efficacy should not be compromised by this new emerging viral strain [26,27].

Altogether, these findings suggest that D3 and Sotrovimab could be used in combinatorial treatments against SARS-CoV-2 infections, especially against the Omicron variant, which is not susceptible to other antibody cocktails [19,24].

In conclusion, we think that D3 mAb could become a precious tool for diagnostic applications in the future pandemic waves of Coronavirus variants on one side and to enhance the therapeutic potential of other therapeutic mAbs, such as Sotrovimab, on the other side. 

## 4. Materials and Methods

### 4.1. Antibodies and Human Recombinant Proteins

Human chimeric SARS-CoV-2 (2019-nCoV) Spike-RBD/Fc, SARS-CoV-2 (2019-nCoV) Spike-RBD/His and ACE-2/His proteins were purchased from Sino Biological, Dusseldorfer Eschborn, Germany. Human chimeric SARS-CoV-2 Omicron-RBD/Fc and IgG1 Fc proteins were purchased from R & D Systems, Minneapolis, MN, USA. Human chimeric ACE-2/Fc and SARS-CoV-2 Omicron-RBD/His proteins (from GenScript, Piscataway, NJ, USA) were also used.

Sotrovimab (from GlaxoSmithKline and Vir Biotechnology), Casirivimab and Imdevimab (from Regeneron Pharmaceuticals) mAbs were used. D3 mAb was expressed and purified as previously described [22]. 

HRP-conjugated anti-His antibody (Proteintech, Deansgate, Manchester, UK), anti-human IgG (Fab’)2 goat polyclonal antibody (Abcam, Banzarate, MI, Italy), anti-human Fc antibody (Sigma, St. Louis, MO, USA), and HRP-conjugated streptavidin (Biorad, Segrate, MI, Italy) were used for the detection of primary mAbs.

Human peptides derived from RBD portion of human SARS-CoV-2 Spike-RBD protein were synthesized from GenScript (Piscataway, NJ, USA) with the following aa sequences:

Peptide 1 Sequence: RKSNLKPFER;

Peptide 2 Sequence: GVEGFNCYFP;

Peptide 3 Sequence: RFASVYAWNRK;

Peptide 4 Sequence: RVQPTESIVR.

### 4.2. ELISA Assays

#### 4.2.1. Binding of D3 and ACE-2 to Spike-RBD Peptides

To test the binding of D3 to human peptides derived from RBD of human SARS-CoV-2, ELISA assays were performed on peptides immobilized on Nunc flat-bottom 96-well plates (Thermo Fisher Scientific, Ferentino, MI, Italy) at the concentration of 5 μg/mL. After the blocking of the plate with 5% nonfat dry milk in PBS for 1 h at 37 °C, D3 mAb or ACE-2/His recombinant protein were added at the concentration of 150 and 300 nM to the plates in 3% Bovine Serum Albumin (BSA Sigma, St. Louise, MO, USA) in PBS and incubated for 2 h at Room Temperature (RT) by gently shaking. After extensive washes, HRP-conjugated anti-His or anti-Fc antibody were added for 1 h at RT for the detection of ACE-2 or D3, respectively. After extensive washes, the plates were incubated with 3,3′,5,5′-tetramethylbenzidine (TMB Sigma-Aldrich, St. Louise, MO, USA) reagent, and the absorbance was measured at 450 nm by the Envision plate reader (Perkin Elmer, 2102, San Diego, CA, USA), as previously reported [22].

To confirm the specificity of binding of D3 mAb for the peptides, ELISA assays were performed also by immobilizing the human recombinant Spike-RBD/Fc protein on a Nunc flat bottom 96-well plate. The binding of D3 mAb to RBD was measured in the absence or in the presence of each single peptide or Spike-RBD used at 20:1 molar excess, as described above.

#### 4.2.2. Binding of ACE-2 Receptor to Wild-Type or BA.1 Omicron Variant of Spike-RBD

To test the binding of the ACE-2 receptor to the parental Spike-RBD protein or to its Omicron variant, parallel ELISA assays were performed on immobilized human recombinant wild-type RBD/Fc or RBD/Fc from Omicron variant (5 μg/mL). The plates were incubated with the recombinant ACE-2/His receptor, used at increasing concentrations (2-20 nM) for 2 h at RT, and after extensive washes, the plates were incubated with the HRP-conjugated anti-His antibody to detect the signal, as described above.

#### 4.2.3. Binding of mAbs to Spike-RBD from Wild-Type SARS-CoV-2 or Its BA.1 Omicron Variant

To compare the binding of D3 and Sotrovimab to the wild-type Spike-RBD protein or to its derived Omicron variant, ELISA assays were performed on immobilized recombinant ACE-2/Fc protein. The plates were pre-incubated with wild-type Spike-RBD/His or Spike-RBD/His from Omicron variant at concentrations of 5 nM or 30 nM for 2 h at RT, and after extensive washes, D3 or Sotrovimab were added at the concentration of 30 nM, and the binding signal was detected by using the HRP-conjugated anti-Fab antibody, as described above [28,29,30,31]. In parallel, the binding of Casirivimab, Imdevimab or an unrelated hIgG, used as a negative control, were tested at the same concentrations.

### 4.3. Competitive ELISA Assays of Biotinylated Sotrovimab

In order to investigate whether D3 and Sotrovimab mAb recognize different epitopes of SARS-CoV-2 Spike-RBD protein, a competitive ELISA assay was performed on ACE-2/Fc recombinant protein immobilized on a Nunc-96-well plate at the concentration of 5 μg/mL. The plate was blocked in 5% nonfat dry milk in PBS for 1 h at 37 °C; then, the recombinant Spike-RBD/Fc wild-type protein was added at the concentration of 5 nM in 3% BSA in PBS buffer for 2 h at RT. After extensive washes, the unlabeled D3, Sotrovimab or Nivolumab (used as unrelated hIgG control) mAbs were incubated at the concentration of 150 nM for 2 h at RT, which was followed by the addition of biotinylated Sotrovimab and left binding for 2 h at RT. After extensive washes, the binding of biotinylated Sotrovimab was detected by using the HRP-conjugated streptavidin, as previously described [22,28].

### 4.4. Interference Assays in Spike/ACE-2 Interaction

Since D3 and Sotrovimab mAbs recognize different epitopes of the Spike-RBD protein, to test their ability to reduce the Spike/ACE-2 interaction, a competitive assay was performed by measuring the binding of ACE-2/His to wild-type or BA.1 Omicron-derived Spike-RBD/Fc protein, in the absence or in the presence of D3 or Sotrovimab, or their combination. To this aim, the two RBD/Fc domains were immobilized in parallel on a Nunc-96-well plate pre-incubated with D3 used at the concentration of 100 nM, Sotrovimab used at the concentration of 50 or 100 nM or their combination in 3% BSA-PBS buffer for 2 h at RT. Then, ACE-2/His was added to the plate at the concentration of 5 or 10 nM for 2 h at RT, and its binding was detected by using the HRP-conjugated anti-His secondary antibody. The following steps were performed as described above.

### 4.5. Cell Cultures

Freshly isolated human nasal epithelial cells were collected by nasal brushing of healthy donors and cultured in PneumaCult (StemCell Technologies, Burnaby, BC, Canada) with 2 mM L-glutamine (Gibco Life Technologies, Paisley, Scotland, UK) and 1% penicillin/streptomycin (St. Louis, MO, USA). Cell cultures were grown at 37 °C as previously described [32].

### 4.6. In Vitro Neutralization of SARS-CoV-2 Variants by D3 mAb

Human primary nasal epithelial cells (3 × 10^5^) [33] were plated in a 6-well plate. D3 antibody was incubated with SARS-CoV-2 viral particles belonging to VOC Delta (0.02) or Gamma (i.e., P.1; 0.3 MOI) at 37 °C. After 2 h, cells were infected with D3-SARS-CoV-2 mixture for 72 h. Non-infected cells were used as the negative infection control. The cells were lysed, and their RNA was extracted. These experiments were performed in a BLS3 authorized laboratory [22].

### 4.7. RNA Extraction and qPCR

Total RNA, isolated from human primary nasal epithelial cells by using TRIzol RNA Isolation Reagent (Thermo Fisher Scientific, Van Allen Way, Carlsbad, CA) was quantified in a NanoDrop™ One/OneC Microvolume UV-Vis Spectrophotometer (Thermo Scientific, Waltham, MA, USA). Reverse transcription was performed with 5× All-In-One RT MasterMix (Applied Biological Materials, Baltic Way, WA, USA), according to the manufacturer’s instructions. The reverse transcription products (cDNA) were amplified by qRT-PCR by using a RT-PCR system (Applied Biosystems, Foster City, CA, USA). The cDNA preparation was analyzed through the cycling method by incubating the complete reaction mix as follows:

○ cDNA reactions: 25 °C for 5 min and 42 °C for 30 min;
○ Heat-inactivation: 85 °C for 5 min;
○ Hold stage: 4 °C.

The targets N and ACTB were detected with the SYBR green approach [34,35] by using BrightGreen 2X qRT-PCR MasterMix Low-ROX (MasterMix-LR; ABM). Human ACTB was used as the housekeeping gene to normalize the quantification cycle (Cq) values of the other genes. These runs were performed on a PCR machine (Quantstudio5, Life-technologies, Paisley, Scotland, UK) with the following thermal protocol:

○ Hold stage: 50 °C for 2 min;
○ Denaturation Step: 95 °C for 10 min;
○ Denaturation and Annealing (×45 cycles): 95 °C for 15 s and 60 °C for 60 s;
○ Melt curve stage: 95 °C for 15 s, 60 °C for 1 min and 95 °C for 15 s.

The details of the primers used in these SYBR green assays are provided below:

N1 Forward (SYBR green): GACCCCAAAATCAGCGAAAT;

N1 Reverse (SYBR green): TCTGGTTACTGCCAGTTGAATCTG;

ACTB Forward (SYBR green): GACCCAGATCATGTTTGAGACCTT;

ACTB Reverse (SYBR green): CCAGAGGCGTACAGGGATAGC.

The relative expression of the target genes was determined by using the 2-ΔΔCq method, as the fold increase compared with the untreated controls, as previously described [22]. The data are presented as means ±SD of the 2−ΔΔCq values (normalized to human ACTB) of three replicates.

### 4.8. Docking Analyses

The D3 antibody was built with the tools available within the ZINC data bank web server and then translated into PDB coordinates. The 3D structure of SARS-CoV-2 RBD-Spike was downloaded from the PDB. The docking experiments were performed with AutoDock Vina.

### 4.9. Statistical Analyses

All the data are given as means ±SD. Statistical significance was defined by unpaired two-tailed Student’s *t*-tests and represented as follows: * *p* < 0.05, ** *p* <0.01, and *** *p* < 0.001. 

## 5. Conclusions

In this study, we investigated the ability of the novel human anti-Spike-RBD D3 mAb, recently isolated in our laboratory and found to be neutralizing for SARS-CoV-2 and its Alpha variant, to exert its anti-viral effects also against other variants of SARS-CoV-2, such as Delta, Gamma and Omicron.

We found that D3 was able to inhibit the infection of Delta and Gamma variants in cell cultures and to efficiently bind to the Omicron variant. Thus, we evaluated the possibility to combine D3 with Sotrovimab mAb, which is the only mAb in clinical use for the treatment of COVID-19 patients affected by the Omicron variant.

We found that D3 and Sotrovimab bind to different epitopes and show additive antagonistic effects in Spike RBD/ACE-2 interactions, suggesting that they could be used in combinatorial treatments against SARS-CoV-2 infections (Omicron variant), not susceptible to other available antibody cocktails.
